# Deepfake tweets classification using stacked Bi-LSTM and words embedding

**DOI:** 10.7717/peerj-cs.745

**Published:** 2021-10-21

**Authors:** Vaibhav Rupapara, Furqan Rustam, Aashir Amaar, Patrick Bernard Washington, Ernesto Lee, Imran Ashraf

**Affiliations:** 1 School of Computing and Information Sciences, Florida International University, Florida, United States of America; 2 Department of Computer Science, Khwaja Fareed University of Engineering and Information Technology, Rahim Yar Khan, Pakistan; 3 Division of Business Administration and Economics, Morehouse College, Atlanta, GA, United States of America; 4 Department of Computer Science, Broward College, Broward County, Florida, United States of America; 5 Information and Communication Engineering, Yeungnam University, Gyeongsan si, Daegu, South Korea

**Keywords:** Deepfake, Deepfake sentiment analysis, Machine learning, Deep learning, Stacked Bi-LSTM

## Abstract

The spread of altered media in the form of fake videos, audios, and images, has been largely increased over the past few years. Advanced digital manipulation tools and techniques make it easier to generate fake content and post it on social media. In addition, tweets with deep fake content make their way to social platforms. The polarity of such tweets is significant to determine the sentiment of people about deep fakes. This paper presents a deep learning model to predict the polarity of deep fake tweets. For this purpose, a stacked bi-directional long short-term memory (SBi-LSTM) network is proposed to classify the sentiment of deep fake tweets. Several well-known machine learning classifiers are investigated as well such as support vector machine, logistic regression, Gaussian Naive Bayes, extra tree classifier, and AdaBoost classifier. These classifiers are utilized with term frequency-inverse document frequency and a bag of words feature extraction approaches. Besides, the performance of deep learning models is analyzed including long short-term memory network, gated recurrent unit, bi-direction LSTM, and convolutional neural network+LSTM. Experimental results indicate that the proposed SBi-LSTM outperforms both machine and deep learning models and achieves an accuracy of 0.92.

## Introduction

The wide proliferation of image and video make devices over the past decade initiated a rapid increase in image and video editing applications and software. Today, a large number and variety of face manipulation software and approaches are available that can manipulate the original faces by placing faces of the user’s choice. Such manipulations are becoming increasingly problematic and cause many social problems, let alone financial losses. Fake videos, audios, and images generated by digital manipulation in particular using deep learning techniques have become a major public concern recently (Westerlund, 2019). The popular term ‘deep fakes’ refer to deep learning-based methods that can generate fake images and videos by replacing the face of a person with the face of another person. Deepfakes leverage powerful techniques from AI (Artificial Intelligence) and specifically make use of deep learning approaches to manipulate audio and visual content. Although primarily aimed at providing entertainment, voice assistants, interactive content for online learning courses, and identity protection, etc., it has become a serious concern for the integrity and privacy of the public. It is disturbing human life as it is used for scamming, defaming notable celebrities, and spreading fake news and malicious hoaxes (Kwok & Koh, 2021). Through social media channels people spreading fake videos and audios of celebrities to fuel revenge. The first and foremost targets of deep fakes are famous personalities, including actors, singers, and politicians, whose faces are transposed onto others without their approval (Pantserev, 2020). Deepfakes are categorized into different types namely photo deep fakes, audio deep fakes, video deep fakes, and audio and video deep fakes. Photo deep fakes, technically known as face and body swapping, are used to replace the face or body of the person with the other. Audio deep fakes are voice spooling techniques where the voices of different persons are interchanged. Video deep fakes are divided into face swapping, face morphing, and full-body puppetry. Audio and video deep fakes is a lip-syncing technique where mouth movements and spoken words spoken are changed in a talking head video.

Sentiment analysis helps to determine people’s sentiments, opinions, attitudes, evaluations, emotions, and appraisals towards entities such as services, products, organizations, events, individuals, topics, issue, and their attributes (Rustam et al., 2019a). Such opinions play an important role in deriving the behavior of people about specific ideas, trends, products, and personalities. With the explosive growth of social media plate forms such as forum blogs, Twitter, and Facebook, etc., people express their views and comments and deep fake technology is no exception. People discuss their opinions about deep fake technology through these platforms. The analysis of such reviews helps to study the mindset and sentiments of people about the deep fake technology. This study formulates the following research questions

• What is the polarity of sentiments found in the gathered data?
• What models perform best for sentiment analysis on deepfake technology?
• Is using Textblob suitable for annotating the data?

For this purpose, this study leverages the use of different machine learning approaches. First, the data related to deep fake are extracted from Twitter using the ‘tweepy’ library (Roesslein, 2009). Then classifiers are applied for training and testing on the preprocessed data. In a nutshell, this study makes the following contributions

• A methodology is proposed to analyze people’s sentiments about the deep fake technology. The proposed methodology involves preprocessing steps and various machine learning and deep learning models are tested. These models include LR (Logistic Regression), ETC (Extra Tree Classifier), GBM (Gradient Boosting Machine), SVM (Support Vector Machines), ADA (AdaBoost) classifier, and GNB (Gaussian Naive Bayes). In addition, deep learning models are used to evaluate their performance in comparison to traditional machine learning classifiers.
• Two feature extraction techniques are tested for their efficacy in sentiment classification. Feature extraction approaches include TF-IDF (Term Frequency-Inverse Document Frequency) and a BoW (Bag of Words).
• A novel approach called SBi-LSTM (Stacked Bi-directional-Long Short Term Memory) is proposed to achieve higher classification accuracy. The performance of these models is analyzed in terms of accuracy, precision, recall, and F1 score. Additionally, the comparison of SBi-LSTM is also made with several state-of-the-art approaches.

The rest of this paper is organized as follows. ‘Related Work’ discusses few research works which are closely related to the current study. The selected dataset, machine learning classifiers, and preprocessing procedure, and the proposed methodology are described in ‘Materials and Methods’. Results are discussed in ‘Results and Discussions’ and finally, ‘Conclusion’ concludes the paper with possible directions for future research.

## Related Work

Sentiment analysis is a data mining approach that deals with people’s opinions through NLP (Natural Language Processing), text analysis, and computational linguistics. There are two major approaches to obtain the sentiments from the given reviews and classify results as positive, negative, or neutral.

### Machine learning based sentiment analysis

Machine learning approaches are easy, simple, and efficient than symbolic approaches. Supervised machine learning is the most common method used for sentiment analysis. Different machine learning algorithms such as ME (Maximum Entropy) classification, NB(Naive Bayes), SVM, DT (Decision Trees), ANN (Artificial Neural Network), k-NN(k-Nearest Neighbor), and ensemble methods are commonly used such as to performs sentiment analysis on movie reviews using NB, SVM, and ME (Pang, Lee & Vaithyanathan, 2002). Similarly, study (Moraes, Valiati & Neto, 2013) presents an ANN-based method for the document-level sentiment classification. The study (Wang et al., 2014) performs a comparative assessment of the achievement of three famous ensemble methods such as boosting, bagging, and random subspace based on the five base learners including NB, DT, ME, k-NN, and SVM. Ensemble methods provide better results than individual and base learners (Onan, Korukoğlu & Bulut, 2016; Lochter et al., 2016). Authors use the Textblob library for preprocessing in Saha, Yadav & Ranjan (2017) and polarity confidence calculation. Using SVM and NB, accuracy scores of 60.1% and 65.2% are obtained from SVM and NB, respectively. The study (Hasan et al., 2018) investigates different techniques used for sentiment analysis by using supervised machine learning approaches such as NB and SVM. Sentiment analysis and the polarity classification are done using Textblob, Sentiwordnet, and W-WSD to find the ratio of positive and negative tweets. Experimental results indicate that Textblob provides better results. Perera & Karunanayaka (2020) performs sentiment analysis using NLP approaches and SVM, NB, and LR are used for this purpose. Tenfold cross-validation is used to validate the results. The obtained accuracy is 78.8%, 76.1%, and 71.5%, for SVM, NB, and LR, respectively.

Besides using simple and single features for text analysis, feature combinations to formulate complex feature vectors help to increase the classification performance. For example, study (Kumar, Harish & Darshan, 2019) conducts analysis on the IMDB (Internet Movie Data Base) movie reviews dataset to identify the sentiment expressed by reviewers. The study uses hybrid features comprising TF-IDF and lexicon features like positive-negative word count. Connotation gives better results both in terms of complexity and accuracy when tested against the classifiers including SVM, NB, k-NN, and ME. Similarly, in Gokulakrishnan et al. (2012) author uses Twitter data for sentiment analysis by using N-gram features with the classifiers namely DT, NB, SMO (Sequential Minimal Optimization), and SVM. By using N-gram as a featured author obtain the best performance and best complexity. The study (Moraes, Valiati & Neto, 2013) compared feature representations for affect analysis including learned n-grams and several manually and automatically crafted affect lexicons. A model named SVRCE (Support Vector Regression Correlation Ensemble) is also proposed to enhance the classification performance which shows better performance than traditional machine learning algorithms.

In addition to single machine learning algorithms, ensemble classifiers tend to show better performance for the task at hand. Ensemble approaches use several base learners to combine their output to form an integrated output for enhancing classification accuracy. For example, in Hasan et al. (2018) the authors use an ensemble technique for sentiment analysis on a Chinese review dataset. By using a stacked approach of SVM, k-NN, and scoring base learners, higher accuracy is achieved. Similarly, Su et al. (2012) uses an ensemble of NB, CB, k-NN, ME, and SVM with N-gram features to perform sentiment analysis on a product review dataset. Along the same lines, adopts a boosting approach with DT as a base learner on N-gram features to achieve higher classification performance. A hybrid approach is used in Anjaria & Guddeti (2014) where ANN, feed-forward SVM, ME, and NB are combined for sentiment analysis.

The study (Alawneh et al., 2021) proposes an approach for sentiment analysis-based sexual harassment detection. The primary goal is to propose an approach that could be utilized towards developing detection systems and enhancing the classification of the different types of malicious human activities by using a machine learning approach. Using TF-IDF with several machine learning algorithms, the study achieves the highest 0.81 accuracy score with stochastic gradient descent and TF-IDF features. Another study (Waheed et al., 2021) performs sentiment analysis for web spam detection using lexicon-based machine learning techniques. Web data and Kaggle data have been used for the experiments and different machine learning models are utilized such as RF, NB, and RCNN (Recurrent Convolutional Neural Networks). The highest accuracy scores of 96.13 and 86.5 are achieved by RCNN on Kaggle and Web data, respectively.

Despite the better performance of machine learning models, labeled training data is required in the supervised machine learning methods for sentiment analysis, and the acquisition of training data is a laborious procedure (Wu, Song & Huang, 2016). On the other hand, unsupervised machine learning approaches do not require the labeled data. Turney (2002) introduces an unsupervised approach to determine the reviews as thumbs-up and thumbs-down. For this purpose, 410 reviews are obtained from opinions and a 74% classification accuracy is achieved. Table 1 shows the overview of related works that utilize machine learning to perform sentiment analysis.

**Table 1:** Selected related work studies in machine learning for sentiment analysis.

Ref.	Features	Classifiers	Dataset
Pang, Lee & Vaithyanathan (2002)	N-gram	NB, ME, SVM	IMDb dataset
Anjaria & Guddeti (2014)	N-gram	NB, SVM, ME, ANN	Twitter datasets
Kumar, Harish & Darshan (2019)	TF-IDF, BoW	SVM, KNN, NB, ME	IMDb movie dataset
Kolchyna et al. (2015)	BoW, TF-IDF, N-gram	NB, DT, SVM (MPQA)	Opinion dataset
Neethu & Rajasree (2013)	N-gram	SVM, NB, ME	Twitter datasets
Gokulakrishnan et al. (2012)	N-gram	DT, NB, SMO, SVM	Twitter datasets

### Lexicon based sentiment analysis

The lexicon-based method calculates the final sentiment values of a review by rating the sentiment tendency of every word or (phrase) in a given review (Saif et al., 2016). Various approaches have been presented which focus mainly on the process of how to assign a score to each sentiment expression. For example in Hu & Liu (2004), negative words are assigned −1, and positive words are assigned +1, the negation words shift the sentiment value. In the study (Taboada et al., 2011), sentiment expressions are assigned from −5 to +5, the 0 is not used, diminishes and intensifiers are handled. The lexicon-based approach is more applicable if insufficient tagged data are available.

A sentiment lexicon is a collection of words or phrases that convey feelings. Each entry in the sentiment lexicon is combined with its sentiment orientation strength and sentiment orientation (Deng, Sinha & Zhao, 2017). Entries in the sentiment lexicon can be categorized into three classes according to their sentiment orientations, which are negative, positive, and neutral. There are several well-known general-purpose constructed sentiment lexicons such as MPQA (Multi-Perspective Question Answering) (Wilson, Wiebe & Hoffmann, 2009), Sentiwordnet (Baccianella, Esuli & Sebastiani, 2010), GI (General Inquirer) (Stone, Dunphy & Smith, 1966), and OL (Opinion Lexicon) (Hu & Liu, 2004). Table 2 summarizes the discussed related works that focus on using ensemble models for sentiment analysis. In this regard, uses features, the ensemble, and its base classifiers, as well as, the dataset used for experiments are discussed.

**Table 2:** Selected related work studies in ensemble learning for sentiment analysis.

Ref.	Features	Ensembles	Base classifiers	Dataset
Wang et al. (2014)	N-gram	Bagging, Boosting, Random Subspace	NB, ME, DT, KNN, SVM	Ten sentiment datasets
Onan, Korukoğlu & Bulut (2016)	N-gram	AdaBoost, bagging, random subspace, and majority voting	NB, LR, SVM and linear discriminant analysis	Nine sentiment analysis datasets from different domains
Tsutsumi, Shimada & Endo (2007)	N-gram	Stacking	ME, SVM	Scoring Movie review dataset
Sarvabhotla, Pingali & Varma (2011)	N-gram, lexicon	SVRCE	SVM	Two web forum datasets
Li, Wang & Chen (2012)	N-gram, lexicon	Stacking	SVM, KNN	Scoring Chinese review dataset
Su et al. (2012)	N-gram	Stacking	NB, CB, KNN, ME, SVM	Three product review datasets
Whitehead & Yaeger (2010)	N-gram	Bagging, Boosting and Random Subspace	SVM	Five product review datasets
Wilson, Wiebe & Hwa (2006)	N-gram, syntactic features	Boosting	DT	MPQA dataset

Additionally, several research works combine the textual and image features for finding the sentiments. For example, Thuseethan et al. (2020) proposed a sentiment analysis framework that carefully fuses the salient visual cues and high attention textual cues are proposed, exploiting the interrelationships between multimodal web data. They stacked multimodal deep association learners to learn the relationships between learned salient visual features and textual features to achieve significant sentiment analysis results on web data. Similarly, another study (Huang et al., 2020) also works on sentiment analysis using the textual and image for sentiment analysis. They proposed a novel method AMGN (Attention-Based Modality-Gated Networks)—to exploit the correlation between the modalities of images and texts and extract the discriminative features for multimodal sentiment analysis.

## Materials and Methods

**Figure 1:**
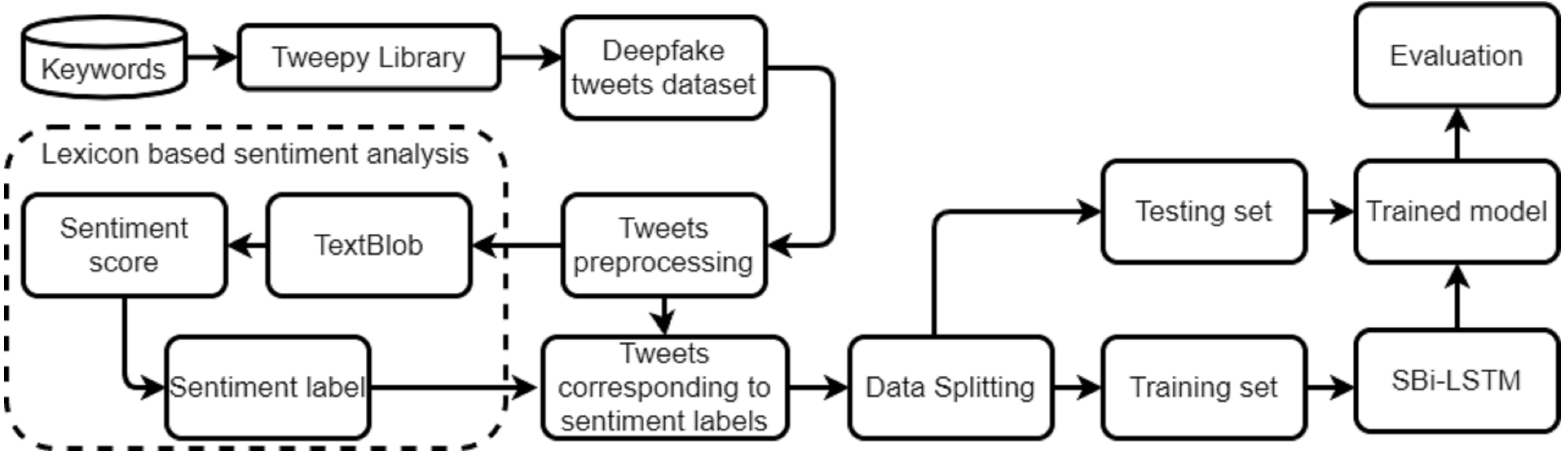
Architecture of the proposed methodology.

This section describes the dataset used for experiments, proposed methodology, and models used for sentiment classification. The flow of the proposed methodology is shown in Fig. 1. In the proposed approach, the dataset is extracted from Twitter using the tweepy library. Afterward, preprocessing is done using the NLP toolkit. Textblob is used for extracting the sentiment from the preprocessed data. Data split is performed in an 85% to 15% ratio for training and testing and performance is analyzed in terms of accuracy, precision, recall, and F1 score.

### Data description

**Figure 2:**
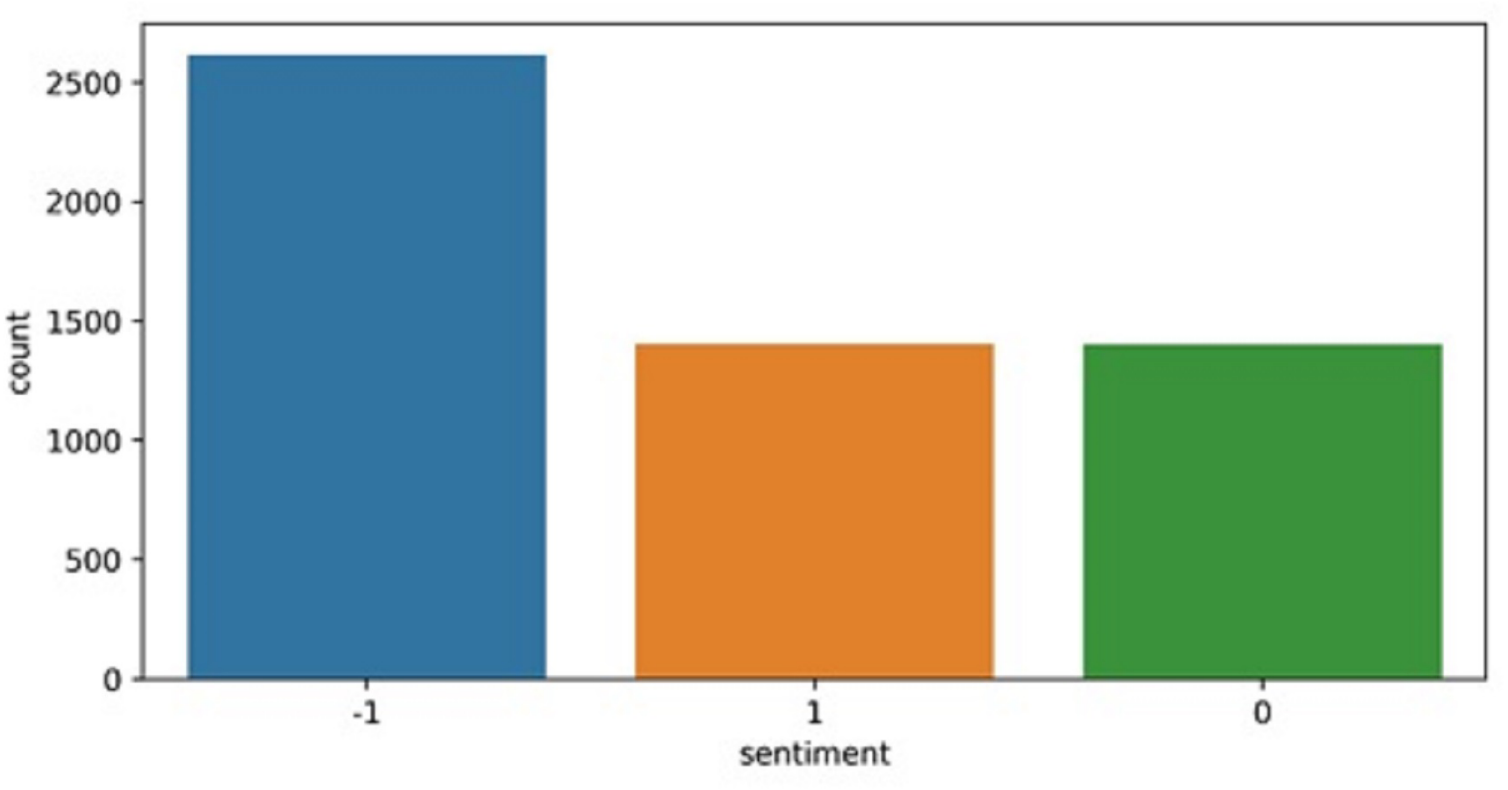
Distribution of negative, positive and neutral tweets in the dataset.

To perform sentiment analysis on deep fake technology, this study extracts the dataset from Twitter. Different keywords are used to extract the tweets such as “# deepfake”, “# deepfakevideo”, and “# deepfaketechnology”. All tweets related to deep fake technology from the last five years (2016 to 2020) are extracted. The extracted tweets contain peoples’ thoughts, opinions, and sentiments about deepfake technology. Such tweets are based on peoples’ experiences in case they become the victims of deepfake, as well as, views on the positive and negative use of deepfake technology. Due to the novelty and lack of knowledge from common people, the number of tweets about deepfake technology is comparatively small. The dataset contains a total of 5,424 tweets and 1,405 as positive, 1,402 are neutral while 2,617 are negative, as shown in Fig. 2. The sentiments of the tweets are extracted using the Textblob. Table 3 shows few sample tweets from the collected dataset.

**Table 3:** Desciption of IMDB dataset variables.

User	Location	Text
pictures_ai	New York	#trump clone will be online for few hours, feel free to live chat on twitch with him: #deeplearning #deepfakeâe—https://t.co/quxwoazwd8
SabineO2010	Leonding, OÃ–	New music from @brendan_m96 on the way!! $\ddot{Y}$Ž¶${\ddot{Y}}^{{^{\prime}}}$$\ddot{Y}$”¥â^3^$\ddot{Y}$ Ž¶#deepfake #NewSingle #cantwait https://t.co/nCFAgEpGtz
minticooki	Chicago, IL	@Fakepix @disclosetv Manipulated. Why is there such desperation to twist and turn photos and print to serve a far râe—https://t.co/v8aKOHz8Eu

### Preprocessing

A large amount of unnecessary data is present in the dataset which plays no important role in the prediction process. Moreover, a large dataset requires a longer time for the training and the stop words directly affect the prediction. So, text preprocessing is required to minimize the computational time, resources and increases accuracy (Zhang et al., 2020). The following steps are carried out in the pre-processing phase.

**Conversion to lower case:** Machine learning models are case sensitive, for example, the model will count the occurrence of the “Deep” and “deep” as two different words. Therefore the first step of the preprocessing is to convert the deep fake data into lower case.

**Removing hashtags, usernames, and punctuation:** In the second step of the preprocessing, hashtags and usernames in tweets are removed. The punctuation marks like, $ % # # & () . ,ˆ ’ ” are removed from the data. These punctuations directly affect the performance because it decreases the ability of an algorithm to distinguish between textual words and these symbols.

**Removing numeric and null values:** Numeric and null values are also removed from the dataset. These values do not play a part in the prediction of the target class. Instead, they increase the feature vector and degrades the performance of classification models. Null values are also regarded as numeric values. Both null and numeric values are removed from the data.

**Removing stopwords:** After the removal of the numeric and null values, the next step is to perform stopwords removal. Stopwords increase the readability of a sentence for human beings, how they are meaningless for classification models.

**Stemming:** Stemming is performed on the text where words are converted to their root/base form. For example, “enjoys” and “enjoyed” words are transformed into their basic form, “enjoy”. So, it is necessary to perform stemming to convert the words into root form.

Table 4 shows the sample tweets from the dataset. The left column shows the original text of the tweet, while the right column shows the processed text after executing all the steps followed in preprocessing.

### Lexicon based sentiment analysis

Textblob is a popular python library for processing textual data (Saad et al., 2021). Textblob provides an API (Application Programming Interface) for NLP tasks. It provides text analysis, text processing, and text mining modules for python developers. Some important features of Textblob include sentiment analysis, tokenization, noun phrase extraction, POS tagging, language translation and detection, n-grams, spelling correction, WordNet integration. Additionally, Textblob is a sentence-level analysis. First of all, it takes data as input, and then it splits the review into sentences. A general way of determining the polarity for entire data is to calculate the number of negative and positive reviews or sentences and judge whether a response is negative or positive based on the total number of negative and positive sentences or reviews.

### Feature selection techniques

The feature selection procedure is carried out to extract the important features from the data to improve the performance of the supervised machine learning models (Rustam et al., 2019b). This process finds the features that correlate with the problem statement which improves the accuracy of the learning models. This study uses two feature selection techniques: TF-IDF and BoW to extract the important features from the data for the training of the models.

#### Term frequency-inverse document frequency

TF-IDF counts the occurrences of unique words in a document and assigns a weight. Weight is calculated for a word that represents its relevancy to that document. The higher the weight of the word is, the more relevant that word will be to that document and vice versa. TF-IDF is calculated by the combination of the two metrics: term frequency (TF) and inverse document frequency (IDF) as shown in Eq. (1). Where TF represents the number of occurrences of a word in the document and assigns higher weights to the higher number of appearances. IDF on the other hand assigns higher weights to those words that are rare and appears less frequently in the document (Yu, 2008). Results of TF-IDF on sample data taken from Table 4 after preprocessing shown in Table 5. Table 5 shows the calculations involved for TF, IDF, and TF-IDF separately to show the difference.

(1) \begin{eqnarray*}tf-idf=T{F}_{t,i}\ast log( \frac{N}{{D}_{t}} ).\end{eqnarray*}

**Table 4:** Tweets before and after preprocessing.

Before preprocessing	After preprocessing
#trump clone will be online for few hours, feel free to live chat on twitch with him: #deeplearning #deepfakeâe— https://t.co/quxwoazwd8	clone online hour feel free live chat twitch
New music from @brendan_m96 on the way!! $\ddot{Y}$Ž¶${\ddot{Y}}^{{^{\prime}}}$$\ddot{Y}$”¥â^3^$\ddot{Y}$ Ž¶#deepfake #NewSingle #cantwait https://t.co/nCFAgEpGtz	music
@Fakepix @disclosetv Manipulated. Why is there such desperation to twist and turn photos and print to serve a far râe— https://t.co/v8aKOHz8Eu	manipulated desperation twist turn photo print

Here, *TF*_*t*,*i*_ is the term frequency of term *t* in tweet *i*. While in IDF *N* is number of tweets and *D*_*t*_ is the total number tweets contain the terms *t*.

#### Bag of Words

The BoW is another widely used technique to extract the features from the text data (Kumar, Harish & Darshan, 2019; Kolchyna et al., 2015; Khalid et al., 2020). It is easy to implement and an easy-to-understand feature extraction technique. For problems like language modeling and text classification, BoW shows remarkable performance. Bow extracts the important features using a count vectorizer. Count vectorizer works similar to term frequency. In BoW, each feature is assigned a value that represents the occurrences of that feature (Hu, Downie & Ehmann, 2009). Results of BoW on sample data taken from Table 4 after preprocessing shown in Table 6.

**Table 5:** Results of TF-IDF on sample data.

Term	TF(D1)	TF(D2)	IDF(D1)	IDF(D2)	TF-IDF(D1)	TF-IDF(D2)
chat	1/8	0/1	log(2/1)	log(2/1)	0.0376	0
clone	1/8	0/1	log(2/1)	log(2/1)	0.0376	0
feel	1/8	0/1	log(2/1)	log(2/1)	0.0376	0
free	1/8	0/1	log(2/1)	log(2/1)	0.0376	0
hour	1/8	0/1	log(2/1)	log(2/1)	0.0376	0
live	1/8	0/1	log(2/1)	log(2/1)	0.0376	0
music	0/8	1/1	log(2/1)	log(2/1)	0	0.301
Online	1/8	0/1	log(2/1)	log(2/1)	0.0376	0
twitch	1/8	0/1	log(2/1)	log(2/1)	0.0376	0

**Table 6:** Results of BoW on sample data.

Doc.	chat	clone	feel	free	hour	live	music	online	twitch
1	1	1	1	1	1	1	0	1	1
2	0	0	0	0	0	0	1	0	0

### Machine learning classifiers

The use of machine learning classifiers for text analysis has produced good results. Consequently, many algorithms and their variants can be found in the literature. For the current study SVM, LR, GNB, ETC, GBM, and ADA are used for deep fake tweets classification. The scikit-learn library is used for the implementation of these algorithms. The performance of these algorithms has been optimized by fine-tuning several hyperparameters. A list of the parameters and their used values that provide the highest accuracy is given in Table 7. A brief description of these algorithms is provided in Table 8.

**Table 7:** Parameters finetuned for machine learning models.

**Algorithm**	**Hyperparameters**
ETC	n_estimators=300, random_state=5, max_depth=300
GBM	n_estimators=300, max_depth=300
LR	solver=’saga’, *C* = 3.0, max_iter=100, penalty=’l2’
SVM	kernel=’linear’, *C* = 2.0, random_state=500
GNB	default setting
ADA	n_estimators=300, max_depth=300, learning_rate=0.2

**Table 8:** Description of machine learning classifiers used in the current study.

Classifier	Description
SVM	SVM is a renowned supervised machine learning algorithm that is widely used for classification and regression problems. SVM performs classification by building high dimensional hyperplanes which are also called decision planes. These hyperplanes help to extricate one type of data from the others (Schölkopf, Burges & Vapnik, 1996).
LR	Most of the classification problems can be usually dealt with using LR. It is a statistical method that carries out predictive analysis using probabilistic inferences. It builds the relationship between the categorical dependent variable and one or more independent variables by approximating the probability by using a Sigmoid function (Boyd, Tolson & Copes, 1987).
GNB	Naïve Bayes has many variants and GNB is one of the most commonly used ones. GNB is used for the continuous data values and encompasses probabilities (posterior and prior) of the classes in the data. GNB assumes that the features are following normal or Gaussian distribution (Perez, Larranaga & Inza, 2006).
ETC	It works very similarly to that of random forest (RF), the only difference lies in the construction of the trees in the forest. Each tree in the ETC is made from the original training sample. Random samples of *k* best values are used for the decision and the Gini index is used to find the top features to split the data in the tree. These random samples of the feature are the indication of the generation of multiple de-correlated decision trees (Sharaff & Gupta, 2019).
GBM	It is a popular machine learning algorithm where many weak classifiers work together to create a strong learning model. GBM works on the principle of the decision trees, however, it creates every tree independently which makes it time-consuming and expensive. It enhances the weak learning algorithms after a series of tweaks which increases the strength of the algorithm. This strength improvement of the algorithms is known as the probability approximately correct (PAC) learning. Due to PAC it works well on the unprocessed data and missing values can be handled efficiently using GBM (Friedman, 2001).
ADA	AdaBoost is the short form of adaptive boosting and it is usually used in combination with the other algorithms to increase their performance. To train weak learners into strong learners, it utilizes the boosting approach. Every tree in Adaboost is dependent on the outcome error rate of the last built tree (Freund, Schapire & Abe, 1999).

### Deep learning approaches

From the last few years, deep learning-based algorithms gained large attention due to the high-rated performance of a variety of tasks. These models can select important features and find their complex relationships to the target class. Deep learning models used in this research are LSTM, GRU, Bi-LSTM, and an ensemble of CNN+LSTM. A brief description of these approaches is provided in Table 9.

**Table 9:** Description of deep learning models used in the current study.

Approach	Description
LSTM	LSTM is a state-of-the-art deep learning technique that is widely used to solve text classification problems. LSTM consists of four gates including input gate, input modulation gate, forget gate, and output gate. All these gates perform different functions. These gates remember the value of the input vector and develop an output vector after looking into the previous history (Tang et al., 2014).
GRU	Like LSTM it has gates, number of gates in the GRU is three which are the current memory gate, reset gate, and update gate. Present input and the previous states are being controlled by these gates. GRU takes current input as the input and previous state as vector and then calculations are performed using these gates (Chung et al., 2014).
Bi-LSTM	Bidirectional LSTM is an extension to the traditional LSTM. Bi-LSTM improves the performance of the model on sequence classification problems. Bi-LSTM is usually used for the problems in which the data is time-stamped for the input sequence. For these scenarios, Bi-LSTM trains two models instead of one LSTM on the input sequence to generate the final results (Schmidhuber, 2015).
CNN+LSTM	Ensemble models tend to show better performance than individual models (Rupapara et al., 2021). The ensemble of CNN+ LSTM has been used largely on account of the advantages of combining the strength of automatic feature extraction in CNN and the capability of capturing the long-term temporal dependencies in LSTM. Consequently, it gives accurate feature representations, which helps the LSTM layers to learn temporal dependencies more precisely. To tackle the time series and classification problems CNN-LSTM is the best choice (Xie, Zhang & Lim, 2020).

### Proposed stacked Bi-directional LSTM architecture

**Figure 3:**
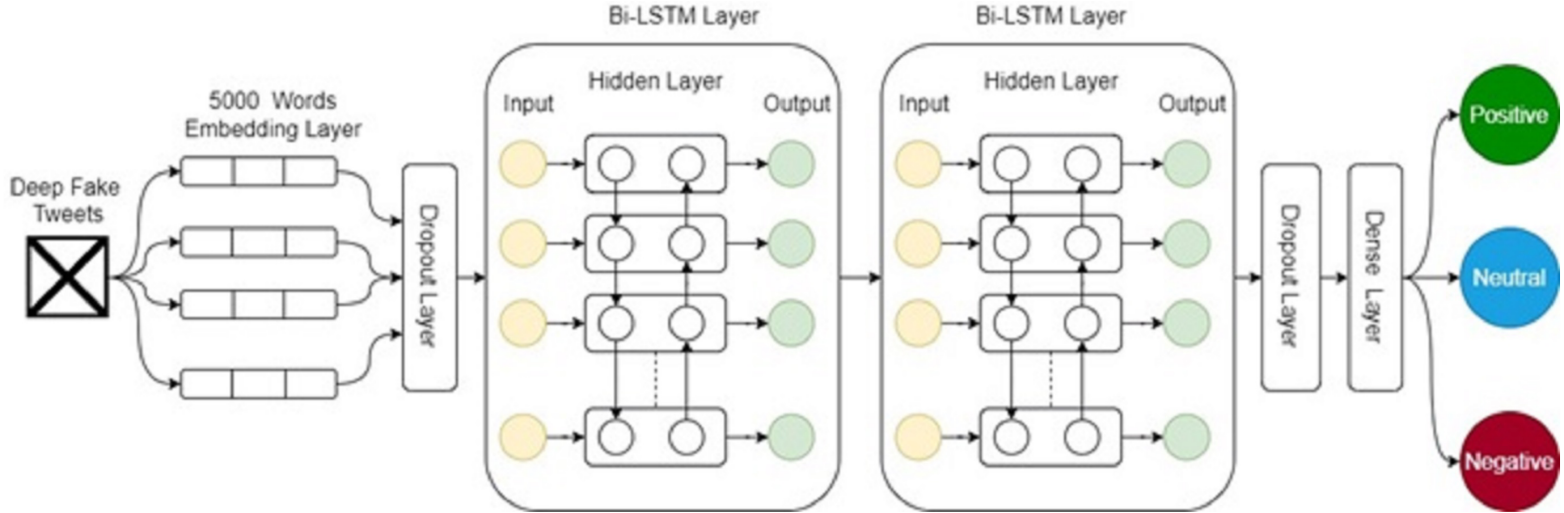
Architecture of the proposed ensemble model.

This study proposes SBi-LSTM for the deep fake sentiment classification and the architecture of the proposed ensemble model is given in Fig. 3. The SBi-LSTM shows better performance as compared to both machine learning and deep learning approaches. The results of SBi-LSTM also reject the hypothesis that deep learning models do not perform well on the small datasets (Rustam et al., 2019a; Rustam et al., 2021b).

The performance of the proposed SBi-LSTM is attributed to its simple structure where multiple layers of LSTM are stacked. It comprises six layers including one embedding layer, two dropout layers, two Bi-LSTM layers, and one dense layer. First, the preprocessed data containing 5,000 words sequences pass to the embedding layer with an output of 100 embedding dimensions (Vo & Hays, 2019). The output of the embedding layer passes through a dropout layer with a 0.5 dropout rate which reduces the complexity at the initial level in input data (Rustam et al., 2021c). Output goes through a stack of Bi-LSTM layers. Bi-LSTM enables additional training by traversing the input data twice (1) left-to-right, and (2) right-to-left). The results show that additional training of data proves to produce better results. The output of the first Bi-LSTM will be input for the second Bi-LSTM to make a more accurate prediction. One dropout layer is used before Bi-LSTMs and one after Bi-LSTMs with a 0.5 dropout layer. In the end, a dense layer is used with a three-unit and a softmax activation function. We compile this model with ‘adam’ optimizer, and ‘categorical_crossentropy’ loss function, and 100 epochs. SBi-LSTM is an ensemble model which outperforms all other models because of its ensemble architecture. The performance of two models joined in ensemble structure can be good as compared to individual models that is the reason SBi-LSTM combines two Bi-LSTM to make a stack. The stacked structure where the first layer finds important features with respect to the target class helps the second layer to provide accurate results. Stacking helps to incorporate the capabilities of well-performing models and make better predictions than a single model. Here, using two Bi-LSTM in a stacked structure helps to achieve better results for sentiment classification. Additionally, it generalizes the model thus increasing the wide use of the proposed approach.

### Evaluation parameters

Accuracy, precision, recall and F1 score are among the most common and widely used performance evaluation parameters (Rustam et al., 2021a). This study uses these parameters to analyze the performance of the machine learning, deep learning and proposed SBi-LSTM model. There are four possible outcomes of the classification models:

• **True positive (TP)**: TP shows the positive predictions of the class that is correctly predicted by the model.
• **True Negative (TN)**: shows the negative predictions of the class that are correctly labeled by the model.
• **False Positive (FP)**: shows the negative predictions of the class that are incorrectly labeled as positive by the classifier.
• **False Negative (FN)**: shows the positive prediction of the class that is incorrectly labeled as negative by the model.

#### Accuracy

It is an important and widely used parameter to evaluate the performance of the models. Accuracy is the ratio between the correctly predicted instances to the total number of predicted instances. It can be calculated by the following formula:

(2) \begin{eqnarray*}Accuracy= \frac{TP+TN}{TP+TN+FP+FN} .\end{eqnarray*}

#### Precision

It is the exactness of the classifier. Precision is the ratio between the positive instances out of total instances which have been predicted positive. It can be calculated by the following formula:

(3) \begin{eqnarray*}Precision= \frac{TP}{TP+FP} .\end{eqnarray*}

#### Recall

The recall is the completeness of the classifier. It shows the percentage of the true positive instances which are labeled correctly. It can be calculated as:

(4) \begin{eqnarray*}Recall= \frac{TP}{TP+FN} .\end{eqnarray*}

#### F1 score

It combines both precision and recall and it is taken as the balanced and well-represented performance of a model. F1 score is the harmonic mean of precision and recall. It can be calculated using

(5) \begin{eqnarray*}F1=2\times \frac{Precision\times Recall}{Precision+Recall} .\end{eqnarray*}

## Results and Discussions

This section presents the results on deep fake tweets using machine learning and deep learning models. Implementation of machine and deep learning models is carried out using Python 3.0 on Jupyter notebook. The performance of machine learning model’s performance in terms of accuracy, precision, recall, and F1 Score. In tables, −1, 0, and +1 represent the negative, neutral and positive sentiment, respectively.

### Results for machine learning models

This section contains the results for machine learning models with both BoW and TF-IDF features. All model’s performance varies according to the feature extraction technique.

#### Results using BoW features

Performance of machine learning models with BoW features is shown in Table 10. Results indicate that the GBM model performs significantly better than other models with a 0.88 accuracy score because of its ensemble boosting architecture. GBM boosts its accuracy even on small data as compared to all other models. SVM is just behind the GBM with a 0.87 accuracy score. This shows that the linear models can also perform better on small data with BoW features. LR and ETC perform equally well with 0.85 accuracy scores.

**Table 10:** Results of machine learning classifiers using BoW features.

Model	Accuracy	Class	Precision	Recall	F1
SVM	0.87	−1	0.92	0.91	0.91
		0	0.76	0.90	0.83
		1	0.91	0.75	0.82
LR	0.85	−1	0.91	0.90	0.91
		0	0.75	0.88	0.81
		1	0.86	0.72	0.78
GNB	0.53	−1	0.67	0.46	0.54
		0	0.66	0.53	0.59
		1	0.38	0.69	0.49
ETC	0.85	−1	0.92	0.90	0.91
		0	0.75	0.93	0.83
		1	0.88	0.70	0.78
GBM	0.88	−1	0.94	0.90	0.92
		0	0.79	0.94	0.86
		1	0.88	0.77	0.82
ADA	0.78	−1	0.88	0.85	0.87
		0	0.75	0.84	0.79
		1	0.64	0.60	0.62

**Figure 4:**
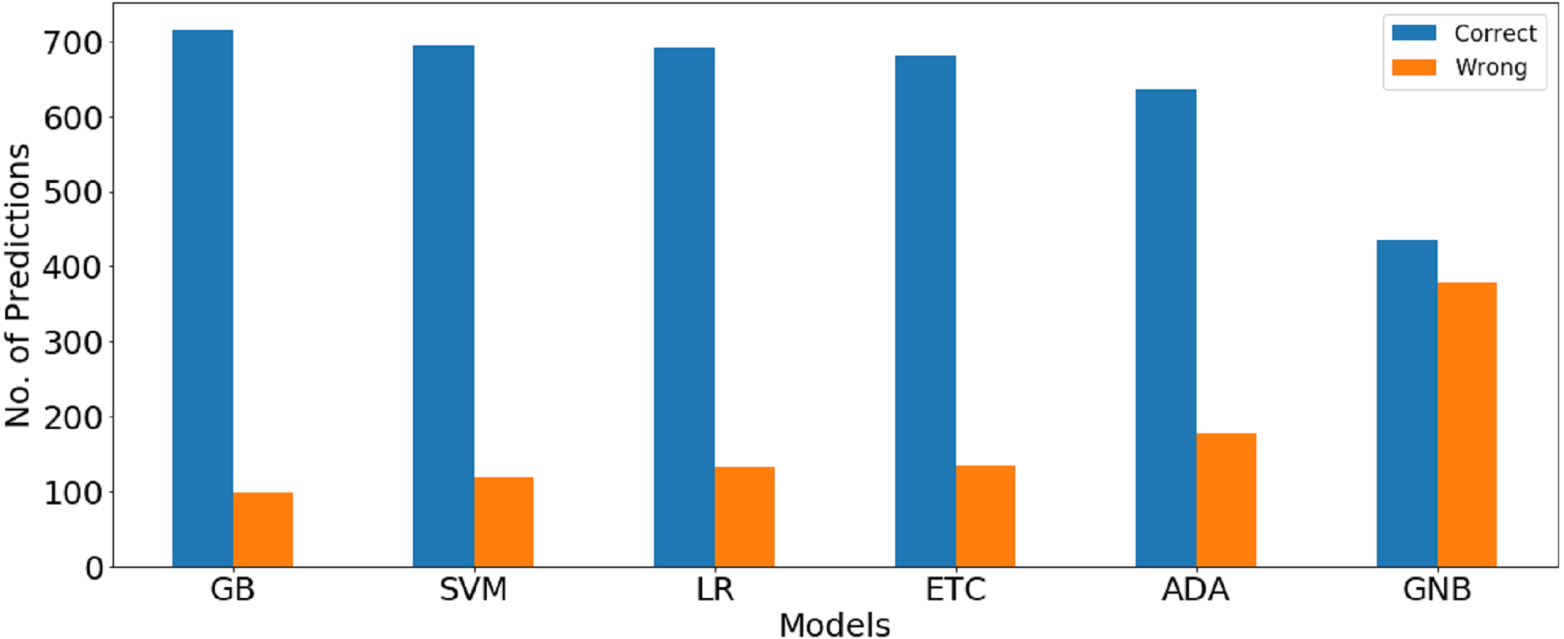
Graphical comparison between number of correct and wrong prediction for machine learning models using BoW features.

The confusion matrix values given in Table 11 show the ratio of the correct and wrong predictions by the machine learning models using BoW features. GBM gives the highest correct prediction with 716 correct predictions out of a total of 814 predictions, whereas 98 predictions are wrong. LR is at second place with 695 correct predictions while GNB gives the lowest correct predictions ratio. Graphical comparison between the number of correct and wrong predictions for machine learning models using BoW features shown in Fig. 4.

**Table 11:** Confusion matrix for machine learning classifiers using BoW features.

Model	Correct predictions	Wrong predictions
SVM	691	123
LR	695	119
GNB	435	379
ETC	680	134
GBM	716	98
ADA	636	178

#### Results using TF-IDF

Results of machine learning models with TF-IDF are shown in Table 12. Results show that the performance of machine learning models has been degraded when used with TF-IDF features. Owing to the small size of the dataset, finding weighted features using TF-IDF does not perform well. Instead, simple term frequency using BoW features provides a better feature vector to train learning models. GBM again outperforms all models in terms of accuracy, precision, recall, and F1 score using TF-IDF features with an accuracy of 0.85. SVM, LR, ETC are behind the GBM with 0.84 accuracies. GNB performs poorly with TF-IDF features as well as with BoW features.

**Table 12:** Results of machine learning classifiers using TF-IDF features.

Model	Accuracy	Class	Precision	Recall	F1
SVM	0.84	−1	0.88	0.90	0.89
		0	0.77	0.85	0.81
		1	0.83	0.71	0.76
LR	0.84	−1	0.89	0.91	0.90
		0	0.77	0.82	0.80
		1	0.82	0.73	0.77
GNB	0.54	−1	0.66	0.48	0.56
		0	0.63	0.54	0.58
		1	0.40	0.65	0.49
ETC	0.84	−1	0.88	0.90	0.89
		0	0.74	0.87	0.80
		1	0.88	0.69	0.77
GBM	0.85	−1	0.93	0.88	0.90
		0	0.76	0.90	0.83
		1	0.83	0.75	0.79
ADA	0.79	−1	0.90	0.87	0.89
		0	0.71	0.83	0.76
		1	0.68	0.60	0.64

**Figure 5:**
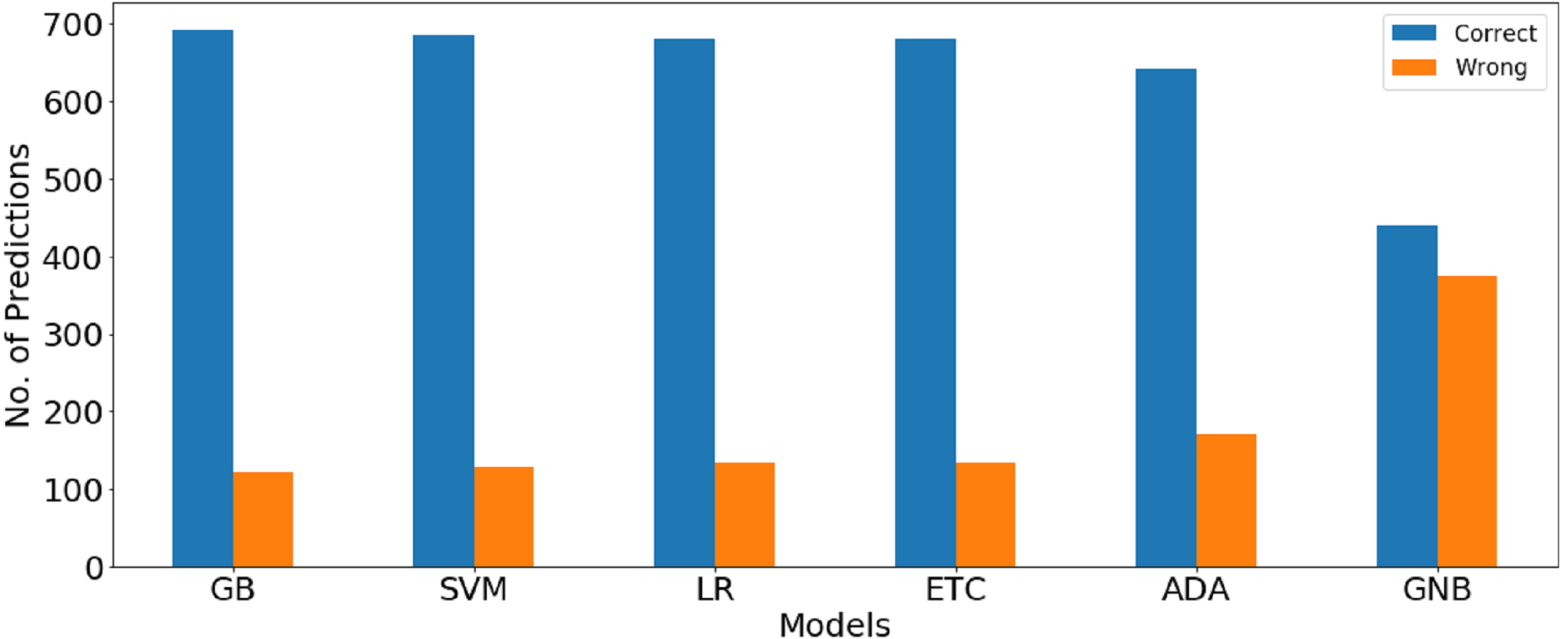
Graphical comparison between number of correct and wrong prediction for machine learning models using TF-IDF features.

Confusion matrix showing correct and wrong predictions using the TF-IDF features is given in Table 13. GBM gives the highest number of correct predictions with 693 correct predictions while GNB shows the worst performance with only 440 correct predictions and the highest wrong predictions of 374. Graphical comparison between the number of correct and wrong predictions for machine learning models using TF-IDF features shown in Fig. 5.

**Table 13:** Confusion matrix for machine learning classifiers using TF-IDf features.

Model	Correct predictions	Wrong predictions
SVM	685	129
LR	681	133
GNB	440	374
ETC	680	134
GBM	693	121
ADA	643	171

### Results of proposed SBi-LSTM model

The performance of the proposed models is significantly better than the machine learning models with an accuracy of 0.92. It also outperforms all other models in terms of precision, recall, and F1 scores with 0.91, 0.88, 0.91 scores, respectively. The performance of the proposed model is due to its simple and stacked architecture. After embedding layer dropout layer reduces the complexity in data and then first Bi-LSTM extracts features for the second Bi-LSTM to generate significant results. It performs equally well on all three target classes as compared to other models. The results of SBi-LSTM are shown in Table 14.

**Table 14:** Results of proposed model SBi-LSTM.

Model	Accuracy	Class	Precision	Recall	F1
SBi-LSTM	0.92	−1	0.94	0.96	0.95
		0	0.89	0.89	0.89
		1	0.91	0.88	0.90

### Performance comparison with deep learning approaches

The performance of the proposed SBi-LSTM model is compared with other deep learning models. The results of all models are shown in Table 15 which indicate that LSTM and GRU provide higher accuracy scores than CNN and ensemble of CNN+LSTM. The performance of CNN and CNN-LSTM is not good because CNN required a large amount of data to show its significance but the used dataset is not large enough which decreases the performance of these models. The proposed SBi-LSTM, on the other hand, shows superior performance and outperforms both machine learning, as well as, deep learning models.

**Table 15:** Comparison of proposed SBi-LSTM with deep learning approaches.

Model	Accuracy	Class	Precision	Recall	F1
CNN	0.62	−1	0.62	0.75	0.68
		0	0.64	0.46	0.54
		1	0.58	0.55	0.57
LSTM	0.81	−1	0.85	0.88	0.86
		0	0.82	0.64	0.72
		1	0.75	0.88	0.81
CNN+LSTM	0.62	−1	0.63	0.73	0.68
		0	0.65	0.46	0.54
		1	0.56	0.60	0.58
GRU	0.81	−1	0.84	0.88	0.86
		0	0.83	0.66	0.73
		1	0.74	0.80	0.77
Proposed	0.92	−1	0.94	0.96	0.95
		0	0.89	0.89	0.89
		1	0.91	0.88	0.90

**Figure 6:**
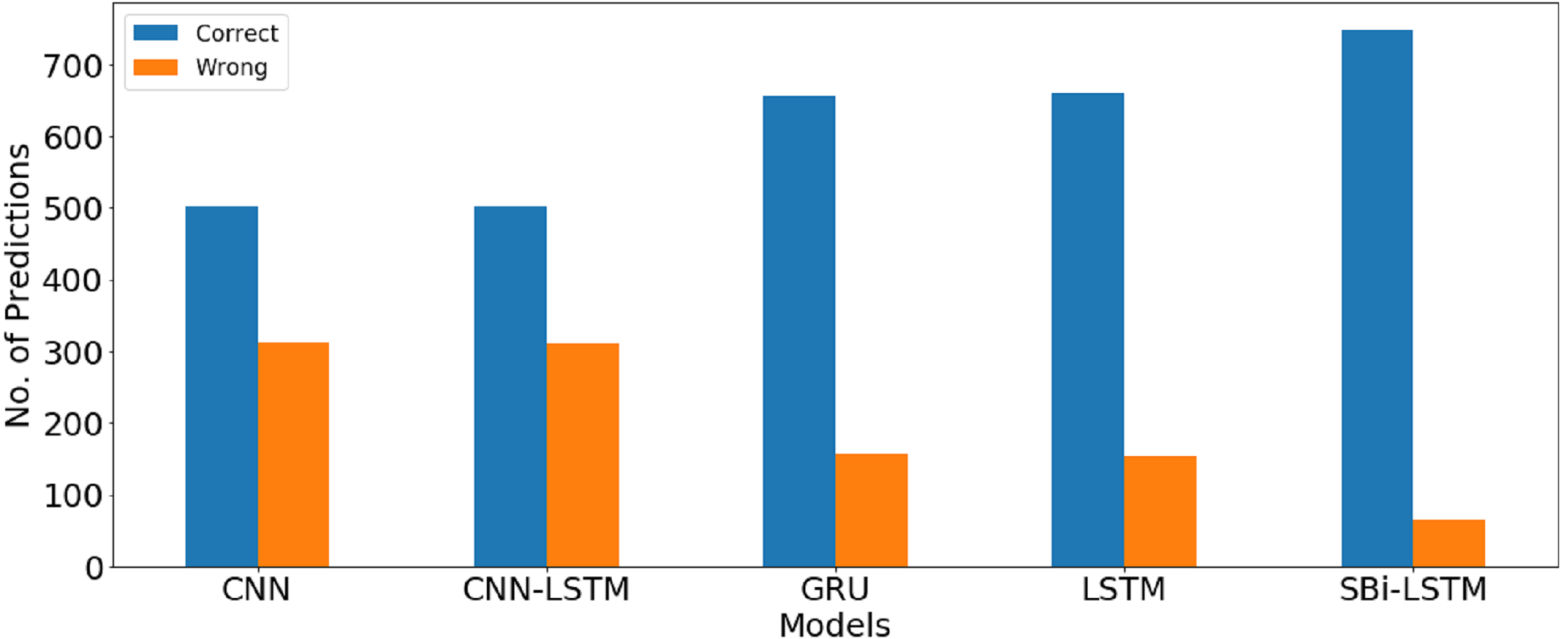
Graphical comparison between number of correct and wrong prediction for deep learning models.

The correct and wrong predictions for all deep learning models are provided in Table 16. Results indicate that SBi-LSTM gives the lowest number of the wrong predictions as compared to all other models which show the significance of the proposed model. The number of correct predictions is 749 while only 62 predictions are wrong. Graphical comparison between the number of correct and wrong predictions for deep learning models shown in Fig. 6.

**Table 16:** Confusion matrix for deep learning classifiers.

Model	Correct predictions	Wrong predictions
CNN	502	312
LSTM	660	154
CNN+LSTM	503	311
GRU	657	157
Proposed	749	62

### The performance of models on US airlines dataset

To show the significance of our approach, additional experiments are performed on another dataset that has been used in Rustam et al. (2019a). We use the US airline dataset, which is publicly available. All the steps of the proposed approach have been performed on this dataset and later state-of-the-art machine learning models are applied in addition to the proposed SBi-LSTM. The results of all models on the US Airline dataset are shown in Table 17 to show the efficacy of the proposed approach for applying it on other datasets.

**Table 17:** The performance of models on US airlines dataset from Rustam et al. (2019a).

Machine learning			Deep learning	
Model	Accuracy		Model	Accuracy
	BoW	TF-IDF		
SVM	0.91	0.91	CNN	0.78
LR	0.91	0.90	LSTM	0.91
GNB	0.38	0.38	CNN+LSTM	0.79
ETC	0.91	0.90	GRU	0.90
GBM	0.90	0.89	SBi-LSTM	0.93
ADA	0.81	0.79		

Machine learning models tend to show better results when BoW feature extraction is used. For example, GBM and SVM achieve the highest accuracy scores of 0.88 and 0.87, respectively with BoW which are reduced to 0.85 and 0.84, respectively, when using TF-IDF features. LR and ETC have marginal degradation from 0.85 each to 0.84 when moved from BoW to TF-IDF. Conversely, ADA and GNB show slightly better performance with TF-IDF achieving accuracy scores of 0.79 and 0.54, respectively against scores of 0.78 and 0.536, respectively with BoW. The difference in the classification performance of deep learning models is substantial with CNN and the ensemble of CNN and LSTM achieving an accuracy score of 0.62 each. GRU and LSTM show better performance with an accuracy score of 0.91 each. The proposed stacked structure shows superior performance than both machine learning and deep learning approaches with 0.92 accuracy. Sequence to sequence learning with bi-directional series of recurrent neural networks is the preferred approach which demonstrates better results than traditional phrase-based approaches. CNN does not depend on previous time step computation and is not commonly used for sequence modeling. Recurrent neural networks maintain the hidden state of the past step and can obtain the context information. CNN has a small training time as compared to LSTM while LSTM can show better accuracy for text classification tasks.

## Conclusion

This study addresses the problem of analyzing the sentiments for deep fake videos using the tweets from Twitter. Data obtained using the tweepy library are used with several machine learning and deep learning models and their performance is evaluated in terms of accuracy, precision, recall, and F1 score. Two well-known feature extractions methods, TF-IDF and BoW, are utilized and their efficacy is tested. In addition, a novel model, SBi-LSTM is proposed which comprises stacked bi-directional LSTM layers where input data is traversed twice to increase its classification accuracy. Results indicate that machine learning classifiers perform better with the BoW features and GBM achieves the highest accuracy of 0.88. Using TF-IDF features, the performance is degraded. On the other hand, the proposed SBi-LSTM performs exceptionally well and obtains a 0.92 accuracy for three classes of the dataset. In contrast to SBi-LSTM, other deep learning models perform poor such as LSTM and GRU with a 0.81 accuracy each and CNN with an accuracy of 0.62. Results of the proposed SBi-LSTM on the US airlines dataset indicate the generalizability of the approach for its application to perform sentiment analysis on data from other domains. The stacked structure is suitable for sentiment analysis on Textblob annotated data from heterogeneous domains. We intend to perform further experiments by increasing the size of the dataset, as well as, incorporating the data from other than the English language in the future.

## Supplemental Information

10.7717/peerj-cs.745/supp-1 Supplemental Information 1 Data files and Code

## Additional Information and Declarations

### Data Availability

The following information was supplied regarding data availability: Data used for experiments and code for implemented models are available as Supplemental Files.
